# Relationship between the Nutritional Status of Vitamin A per Trimester of Pregnancy with Maternal Anthropometry and Anemia after Roux-en-Y Gastric Bypass

**DOI:** 10.3390/nu9090989

**Published:** 2017-09-08

**Authors:** Sabrina Cruz, Andréa Matos, Suelem Pereira da Cruz, Silvia Pereira, Carlos Saboya, Andréa Ramalho

**Affiliations:** 1 School of Medicine, Federal University of Rio de Janeiro (UFRJ), Rio de Janeiro 21.941-902, Brazil; amatosnut@gmail.com (A.M.); suelemcruz.med@gmail.com (S.P.d.C.); se.pereira@gmail.com (S.P.); 2 Center for Research on Micronutrients (NPqM), Institute of Nutrition Josué de Castro of UFRJ, Rio de Janeiro 21.941-902, Brazil; cjsaboya@carlossaboya.com.br (C.S.); aramalho.rj@gmail.com (A.R.); 3 Fluminense Federal University (UFF), Rio de Janeiro 24.020-150, Brazil; 4 Multidisciplinary Center for Bariatric and Metabolic Surgery, Rio de Janeiro 22280-020, Brazil; 5 Escola Paulista de Medicina, Federal University of São Paulo (UNIFESP), São Paulo 04.021-001, Brazil; 6 Fundação Oswaldo Cruz (ENSP/FIOCRUZ), Rio de Janeiro 21040-900, Brazil; 7 Department of Social and Applied Nutrition of the Institute of Nutrition, UFRJ, Rio de Janeiro 21.941-902, Brazil

**Keywords:** pregnancy, Roux-en-Y gastric bypass, bariatric surgery, vitamin A, anemia

## Abstract

The aim of this study was to compare the nutritional status of vitamin A per trimester of pregnancy, as well as to assess its influence on pre-pregnancy BMI, total gestational weight gain (TGWG) and presence of anemia in women who had previously undergone Roux-en-Y gastric bypass (RYGB). An analytical, longitudinal and retrospective study comprising 30 pregnant women who had previously undergone RYGB was undertaken. In all trimesters of pregnancy, the serum concentrations of retinol, β-carotene, stages of vitamin A deficiency (VAD), night blindness (NB), anemia and anthropometric variables were assessed. VAD in pregnancy affected 90% of women, 86.7% developed NB and 82.8% had mild VAD. TGWG above/below the recommended range was related to the low serum concentrations of β-carotene (*p* = 0.045) in the second trimester and women with TGWG above the recommended range showed 100% of inadequacy of this nutrient in the third trimester. Among the pregnant women with anemia, 90.9% had VAD and 86.4% had NB. This study highlights the importance of monitoring the nutritional status of vitamin A in prenatal care, due to its relationship with TGWG and the high percentage of VAD and NB found since the beginning of pregnancy. It also reaffirms the use of the cut-off <1.05 μmol/L for determining VAD.

## 1. Introduction

At present, there is an exponential increase of individuals undergoing bariatric surgery [1]. Women with class III obesity account for 73–80% of these patients and 43% are of reproductive age [2]. Bariatric surgery reduces associated diseases, modifies the regularity of menstrual cycles, increases fertility and thereby favors the occurrence of pregnancy [3,4].

For the last decade, the most performed surgical procedure worldwide has been Roux-en-Y gastric bypass (RYGB), able to provide health benefits [5]. However, the resulting metabolic disorders can trigger important nutritional deficiencies such as vitamin A deficiency (VAD), considered a serious public health problem that affects 19 million pregnant women globally [6,7,8].

It is acknowledged that pregnancy impacts on the inadequacy of serum vitamin A since its requirements increase by 40% for the maintenance of placenta and fetal development, mainly in the third trimester when the greatest demand for this nutrient occurs [9,10,11]. In the literature, only one study addressing post-bariatric pregnant women reported that the greatest inadequacy of vitamin A occurs at the end of pregnancy. However, questions remain to be clarified since the study assessed different surgical procedures [12].

Another factor that contributes to VAD is an increase in tissue adiposity [13]. In this context, the permanence of overweight and/or obesity in pre-pregnancy, a frequent occurrence after bariatric surgery, and gestational weight gain above the recommended range, are considered determining factors for gestational adverse outcomes [1,4,14,15] and can still contribute to the worsening of the nutritional status of vitamin A.

Moreover, it has been reported that women who had undergone bariatric surgery prior to pregnancy have higher chances of developing anemia when compared to pregnant women who had not undergone this surgery [16]. In this respect, the literature shows that pregnant women with VAD are 2.7 times more likely to develop anemia when compared to women without deficiency of this vitamin and that hemoglobin concentrations decreased by 95.7% in women who had VAD in pregnancy [17].

In this way, we intend to compare the nutritional status of vitamin A among women who had undergone bariatric surgery prior to pregnancy in different trimesters of pregnancy, as well as to assess the influence of pre-pregnancy BMI and/or gestational weight gain on serum concentrations of vitamin A during pregnancy and the relationship between anemia and VAD per trimesters of pregnancy.

## 2. Methodology

This is an analytical, longitudinal and retrospective study comprising adult women of reproductive age, aged 22–39 years, who had previously undergone RYGB, carried out at the Multidisciplinary Center of Bariatric and Metabolic Surgery in the municipality of Rio de Janeiro from January 2011 to July 2015.

**Figure 1:**
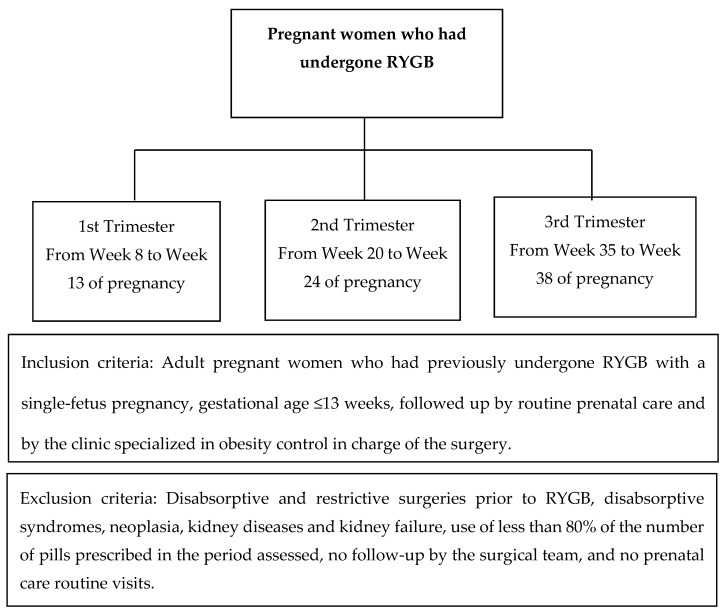
Description of postoperative times, inclusion and exclusion criteria.

The women who had undergone RYGB and had met the inclusion and exclusion criteria set up by the research were assessed over the three trimesters of pregnancy, as described in Figure 1.

Throughout the postoperative follow-up in the Multidisciplinary Center, women were recommended to inform the medical team if they become pregnant so that assistance could be provided during pregnancy in addition to the assistance provided by the routine prenatal care outside the Center. In general, they returned between Week 8 and Week 13 of pregnancy. All participants followed a routine protocol with multivitamin and mineral supplementation containing 5000 IU of retinol acetate, 90 mg of iron and 6 mg of folic acid [18].

For the assessment of adherence to the proposed supplementation, the containers of the prescribed supplements were requested in all consultations in which the importance of their daily use was emphasized and educational materials showing the resulting beneficial effects were delivered.

For anthropometric assessment, data were collected on height, pre-pregnancy weight informed or measured until Week 13 of pregnancy, pre-delivery weight, or weight recorded in the last prenatal consultation, and pre-surgery weight. These variables were used to calculate and classify BMI according to the cut-off points set by the World Health Organization (WHO, 1998) [19]. Besides, these data were used to calculate the total gestational weight gain (TGWG) by subtracting the pre-pregnancy weight from the pre-delivery weight. Gestational weight gain adequacy was classified in accordance with the Institute of Medicine recommendation (2009) that considers a 5–9 kg increase of body weight as appropriate [20].

Information on the presence of anemia was analyzed during the trimesters of pregnancy with cut-off points of serum hemoglobin less than 11.0 g/dL in accordance with the Centers for Disease Control and Prevention recommendations (1998) [21].

For the biochemical assessment of vitamin A, retinol and β-carotene were dosed by the method of high performance liquid chromatography with UV detector (HPLC-UV) over the trimesters. The following cut-off points were used to indicate inadequacy: serum values of retinol <1.05 μmol/L (<30 μg/dL) and β-carotene ≤40 µg/dL [22]. In addition, retinol concentrations were measured at class intervals and the resulting classification of VAD was as follows: severe (>0.35 μmol/L), moderate (≥0.35–0.70 μmol/L) or mild (≥0.70–1.05 μmol/L) [23].

For functional assessment, the presence of night blindness (NB) was investigated in all trimesters of pregnancy through a standardized interview set by WHO (1996) and the Pan-American Health Organization (PAHO) [24], adapted and validated for pregnant women by Saunders and co-workers [25,26].

The instrument used for data collection was pre-tested and consisted of a form filled out by a single interviewer through an interview and analysis of the pre-natal medical records, which was complemented in the nutritionist consultation.

For statistical analysis, the Shapiro–Wilk tests were used for checking normality. Quantitative data were described in measures of central tendency and dispersion. For comparison of means, the Mann–Whitney test or the Kruskal–Wallis were used. For testing the homogeneity of proportions between categorical variables, the Chi-square test was applied. In the analyses, a 5% significance level was adopted. All statistical assessment was carried out using the statistical software SPSS for Windows version 21.0.

## 3. Results

### 3.1. Sample Characterization

The study was conducted with 30 pregnant women in reproductive age who had previously undergone RYGB, mean age of 30.33 ± 4.38 years, minimum of 22 and maximum of 39 years, with a mean interval of 17.70 ± 9.07 months between the last menstrual period (LMP) and surgery. Pre-surgery BMI comprised individuals with class II or class III obesity with mean of 43.52 ± 5.79. The highest percentages were found in class III obesity with 86.7% of the cases. Mean pre-pregnancy BMI was 27.36 ± 3.26, with 24.7% classified as normal weight, 55.2% as overweight and 20.7% as class I obesity. During the trimesters of pregnancy, mean BMI presented a discreet increase, as shown below: first trimester: 27.98 ± 3.13; second trimester: 28.95 ± 3.13; third trimester: 30.14 ± 3.43. As for TGWG adequacy, 34.5% of the women presented appropriate weight gain, 13.8% were above the recommended weigh gain and 51.7% were below.

### 3.2. Biochemical and Functional Assessment of Vitamin A

In this study, VAD affected 90% of the pregnant women and 86.7% developed gestational NB. Among women who developed gestational VAD, 88.9% presented NB: 59.3% developed NB in the first trimester, 59.3% in the second and 65.4% in the third trimester, with no significant difference found between the assessed periods (*p* = 0.870). Furthermore, an association was found between inadequacy of retinol and presence of NB in the second trimester (*p* = 0.002).

Mean β-carotene was below the cut-off point for adequacy regardless of the trimester of pregnancy, while retinol had adequate means in the second and the third trimesters without significant differences throughout pregnancy, albeit the percentage of inadequacy of both nutrients remained similar in each trimester (Table 1).

In addition, no significant differences were found between serum means and percentage of inadequacy of retinol and β-carotene throughout the trimesters of pregnancy when pregnant women were classified according to their pre-pregnancy BMI (normal weight vs. overweight vs. class I obesity, or normal weight vs. overweight). Nevertheless, it is worth pointing out that pre-pregnancy BMI of women with normal weight presented higher percentages of inadequacy of retinol and β-carotene in the second and the third trimesters, while those overweight or obese had higher percentages of inadequacy in the first trimester (Table 2).

Women with TGWG above and/or below the recommended weight showed percentages of inadequacy of β-carotene in the second trimester significantly higher when compared to those with adequacy (*p* = 0.032). It is also worth pointing out that those women who presented TGWG above the recommended range had a lower percentage of inadequacy of retinol in the third trimester; however, β-carotene was 100% inadequate (Table 3).

### 3.3. Biochemical and Functional Assessment of Vitamin A According to Severity

When classifying VAD according to severity, higher percentages were observed in the mild level with 82.8% of the total cases with the following distribution: 56.7% in the first trimester, 70% in the second trimester, 65.5% in the third trimester (*p* = 0.334), and 6.7% with moderate severity in the first trimester. In addition, association was found between the serum inadequacy of retinol and presence of NB in the first and second trimesters in women with mild VAD (*p* = 0.041 and *p* = 0.028, respectively).

When classifying VAD according to severity considering the TGWG, it was observed that women with appropriate weight gain had higher percentages of mild VAD with 50%, 60% and 80% of involvement in the first, second and third trimester of pregnancy (*p* = 0.459), respectively, and 10% of moderate VAD in the first trimester. As regards women with TGWG below adequacy, mild VAD was present in higher percentages in the last two trimesters with 60%, 80%, 66.7% (*p* = 0.547), and 6.7% developed moderate VAD in the first trimester. As regards women with TGWG above the recommended range, mild VAD was higher in the first two trimesters with values reaching 50%, 75%, and 25% (*p* = 0.368) per trimester of pregnancy.

It is also highlighted that NB was present in 100% of women who showed weight above the recommended range, while in those with appropriate weight gain and in those with weight gain below the recommended range the percentage of NB found was 80% and 86.7%, respectively.

### 3.4. Relationship between the Presence of Anemia and Vitamin A

Of the women assessed, 73.3% developed anemia in pregnancy. The mean values of hemoglobin showed a significant difference during pregnancy since its mean concentrations were adequate in the first trimester (11.05 ± 1.22) and inadequate in the second (10.82 ± 0.65) and third trimesters (10.79 ± 0.87) with *p* < 0.001. However, the percentage of women affected with anemia per trimester of pregnancy was similar: in the first trimester =46.7%, in the second =30% and in the third =55.2 with *p* = 0.139. Among pregnant women with anemia, 90.9% presented VAD and 86.4% NB, but no association was found between the presence of anemia and VAD in pregnancy.

## 4. Discussion

Serum concentrations of retinol tend to decrease during the trimesters of pregnancy and serum levels are intensely required in the last trimesters when compared to the first trimester [11]. Such a scenario can be exacerbated if the pregnant woman had previously undergone bariatric surgery. The disabsorptive and restrictive components of most surgical procedures can contribute to a reduction of vitamin A absorption sites, can favor lower food intake and decrease the absorption of lipids and consequently reduce the absorption of fat-soluble vitamins. Such aspects contribute to a decrease in serum levels of retinol [12].

Despite this, the present study shows that the means of the serum concentrations of retinol reached adequacy in the last two trimesters of pregnancy, while β-carotene was below the cut-off point during the entire pregnancy. This may have occurred since β-carotene is required to maintain the serum concentrations of retinol through its bioconversion [27].

According to the World Health Organization (2014), NB is the first functional manifestation of VAD and its greatest prevalence occurs with increasing severity of this vitamin deficiency (serum concentrations <0.7 µmol/L) [28]. Nonetheless, in our study, we observed that over 80% of pregnant women developed mild VAD (0.70 ≤ VAD < 1.05 μmol/L) and an association was found between serum inadequacy of retinol and presence of NB in the first two trimesters of pregnancy. Similar results were found in pregnant women without prior bariatric surgery in whom NB affected 38.5% of the women classified as having mild VAD [26].

From these results, it becomes clear that pregnant women can develop NB with serum levels of retinol ranging from 0.70 to 1.05 μmol/L and that those women who had previously undergone RYGB present higher percentage of this functional change, diagnosed at the beginning of pregnancy and with no significant differences found during pregnancy, thereby confirming that the symptom may appear in the presence of physiologically acceptable circulating levels of retinol.

The presence of NB can be related to maternal and child adverse outcomes, with highlights to pregnant women exposed prior to the surgical procedure [25,26,29]. In this regard, it is worth pointing out the excess risk of persistent NB in pregnant women with serum levels of retinol ranging from 0.70 to 1.05 μmol/L [24,25,26,30], reinforcing the use of the cut-off point of <1.05 μmol/L to determine inadequacy of VA in pregnant women.

Some studies report inverse relationship between BMI and serum concentrations of retinol in individuals with obesity [31,32,33], including serum concentrations of carotenoids that can be 4.5 times smaller in pregnant women with obesity when compared to those with normal weight [34]. In this sense, it may be noted that excess body weight, per se, can contribute to inadequacy of vitamin A.

Thus, the literature points out that excess adipose tissue can also increase VAD since it can influence vitamin A homeostasis by expressing retinol-binding protein in high concentrations, with its retinol reserves stored, metabolized and mobilized to meet the body’s demands [35,36]. Thus, vitamin A may be required by adipocytes or be dissipated passively by the stored lipids [13,37,38], further contributing to decrease its serum concentrations.

However, when the concentrations of retinol and β-carotene per trimesters of pregnancy were compared, it was observed that pregnant women who had previously undergone RYGB had inadequate serum concentrations of retinol or near inadequacy, regardless of the pre-pregnancy BMI range. However, pregnant women with pre-pregnancy BMI featuring overweight and/or obesity had higher percentages of inadequacy both of retinol and β-carotene in the first trimester of pregnancy, thus highlighting the importance of adequacy of vitamin A since the beginning of pregnancy.

According to a recent review, TGWG above or below the recommended range has been associated with adverse maternal and child outcomes, such as prematurity [39]. However, no studies were found relating VAD to TGWG in post-bariatric pregnant women.

In this context, the current study shows that women with TGWG above and/or below the recommended range presented inadequacy of β-carotene significantly higher in the second trimester when compared to those classified as having appropriate TGWG. Besides, this study shows that women with TGWG above the recommended range presented 100% of inadequacy of this nutrient in the third trimester. This result highlights the importance of appropriate weight gain during pregnancy for possible maintenance of the serum concentrations of vitamin A.

A recent review has shown that VAD in pregnancy, in addition to increasing the risk of NB, contributes to the development of anemia and can cause congenital malformations [40]. According to the WHO, anemia can affect 41.8% of pregnant women worldwide [41]. In addition, a positive correlation between the concentrations of retinol and hemoglobin has been found in pregnancy [17].

Of the participants assessed, 73.3% developed anemia in pregnancy, which is a recurring symptom after bariatric surgery [42,43,44] that can be worsened by gestational hemodilution [45]. However, no association was found between the presence of anemia and VAD in our findings, but, among the patients with inadequate hemoglobin, over 85% developed NB and VAD. Thus, we suggest a further investigation of anemia in women with NB or VAD in prenatal care.

The current study presents some limitations, such as those related to the number of patients assessed and the non-inclusion of a control group. However, studies that jointly address the relationship of VAD, according to severity of the deficiency, with maternal anthropometry in pregnant women who had previously undergone RYGB have not yet been found in the literature.

## 5. Conclusions

We highlight the importance of monitoring the nutritional status of vitamin A in prenatal care from the beginning of pregnancy for women who had previously undergone RYGB, in view of the high percentage of VAD and NB found starting in the first trimester of pregnancy. Our data reaffirm the relationship between mild VAD and the presence of NB, pointing out the use of the cut-off point of <1.05 μmol/L to determine the deficiency of this vitamin in the segment assessed in our study.

This study also found that pre-pregnancy BMI did not relate to the high percentage of inadequacy of retinol and β-carotene in the assessed trimesters and that the TGWG above/below the recommended range was related to low serum concentrations of β-carotene.

Given the high percentage of anemic pregnant women with VAD and NB, we suggest a further investigation of anemia in women with changes in the nutritional status of vitamin A in prenatal care.

## Figures and Tables

**Table 1:** Mean serum concentrations of retinol and β-carotene and percentage of night blindness, per trimesters of pregnancy.

	1st Trimester	2nd Trimester	3rd Trimester	*p*-Value
Retinol				
Mean/Standard deviation	0.99 ± 0.29	1.07 ± 0.18	1.09 ± 0.31	0.266
% of Inadequacy	63.3	63.3	65.5	0.886
β-Carotene				
Mean/Standard deviation	31.06 ± 16.12	32.83 ± 16.12	31.03 ± 16.03	0.871
% of Inadequacy	80.0	66.7	75.9	0.643
Night Blindness				
% Present	56.7	56.7	58.6	0.708

The Kruskal–Wallis test was used for continuous variables and the Chi-Square test for categorical variables (*p* < 0.05).

**Table 2:** Percentage of inadequacy of retinol and β-carotene in different classes of pre-pregnancy BMI, per trimesters of pregnancy.

Retinol	1st T	2nd T	3rd T	*p*-Value	β-carotene	1st T	2nd T	3rd T	*p*-Value
Normal weight	42.9	85.7	71.4	0.223	Normal weight	85.7	85.7	100	0.575
Overweight	62.5	56.3	68.8	0.766	Overweight	75	62.5	62.5	0.687
Class I Obesity	85.7	57.1	50	0.424	Class I Obesity	85.7	57.1	83.3	0.398
*p*-value	0.249	0.373	0.663		*p*-value	0.765	0.460	0.137	
Normal weight	42.9	85.7	71.4	0.223	Normal weight	85.7	85.7	100	0.575
Overweight	69.6	56.5	60.9	0.656	Overweight	78.3	60.9	68.2	0.440
*p*-value	0.372	0.215	1		*p*-value	0.66	0.372	0.087	

The Kruskal–Wallis test was used for continuous variables and the Chi-Square test for categorical variables (*p* < 0.05); T = trimester.

**Table 3:** Percentage of inadequacy and means of serum concentrations of retinol and β-carotene according to total gestational weight gain, per trimesters of pregnancy.

TGWG	Retinol			*p*-Value	β-carotene			*p*-Value
	1st T	2nd T	3rd T		1st T	2nd T	3rd T	
Adequate	0.92 ± 0.21	1.05 ± 0.13	1.04 ± 0.19	0.387	35.20 ± 18.28	40.20 ± 18.18	37.60 ± 17.86	0.741
Below	1.02 ± 0.37	1.07 ± 0.22	1.12 ± 0.22	0.549	29.46 ± 14.11	27.80 ± 12.83	28.33 ± 16.58	0.627
Above	1.06 ± 0.15	1.11 ± 0.19	1.09 ± 0.07	0.819	24.5 ± 20.69	31 ± 20.28	24.75 ± 4.03	0.472
*p*-value	0.779	0.472	0.097		0.779	0.368	0.097	
	% Inadequacy of retinol			*p*-value	% Inadequacy of β-carotene			*p*-value
	1st T	2nd T	3rd T		1st T	2nd T	3rd T	
Adequate	60	50	80	0.366	70	40	60	0.387
Below	66.7	73.3	66.7	0.897	86.7	86.7	80	0.844
Above	50	75	25	0.368	75	75	100	0.549
*p*-value	0.819	0.442	0.146		0.586	0.045 *	0.248	

The Kruskal-Wallis test was used for continuous variables and the Chi-Square test for categorical variables (* *p* < 0.05); T = trimester; TGWG = total gestational weight gain.

## References

[1] González I., Rubio M.A., Cordido F., Bretón I., Morales M.J., Vilarrasa N., Monereo S., Lecube A., Caixás A., Vinagre I. (2015). Maternal and perinatal outcomes after bariatric surgery: A Spanish multicenter study. Obes. Surg..

[2] Benoit S.C., Hunter T.D., Francis D.M., De La Cruz-Munoz N. (2014). Use of bariatric outcomes longitudinal database (BOLD) to study variability in patient success after bariatric surgery. Obes. Surg..

[3] Adeboye B., Bermano G., Rolland C. (2012). Obesity and its health impact in Africa: A systematic review. Cardiovasc. J. Africa.

[4] Chaichian S., Moazzami B., Jesmi F., Pazouki A., Pishgahroudsari M., Mokhber S., Riazi S. (2016). The controversy of the most proper time for pregnancy after bariatric surgery: A review of ten cases. Obes. Surg..

[5] Buchwald H., Oien D.M. (2013). Metabolic/bariatric surgery worldwide 2011. Obes. Surg..

[6] Fandiño J., Benchimol A.K., Coutinho W.F., Appolinário J.C. (2004). Cirurgia bariátrica: Aspectos clínico-cirúrgicos e psiquiátricos. Rev. Psiquiatr..

[7] Bloomberg R.D., Fleishman A., Nalle J.E., Kini S. (2005). Nutritional deficiencies following bariatric surgery: What have we learned?. Obes. Surg..

[8] Organização Mundial Da Saúde (2011). Vitamin and Mineral Nutrition Information System (Vmnis). http://www.who.int/vmnis/en/index.html.

[9] Thorne-Lyman A.L., Fawzi W.W. (2012). Vitamin A and carotenoids during pregnancy and maternal, neonatal and infant health outcomes: A systematic review and meta-analysis. Paediatr. Perinat. Epidemiol..

[10] Jans G., Matthys C., Bogaerts A., Lannoo M., Verhaeghe J., Van der Schueren B., Devlieger R. (2015). Maternal micronutrient deficiencies and related adverse neonatal outcomes after bariatric surgery: A systematicreview. Adv. Nutr..

[11] Yang C., Chen J., Liu Z., Yun C., Piao J., Yang X. (2015). Prevalence and influence factors of vitamin A deficiency of Chinese pregnant women. Nutr. J..

[12] Devlieger R., Guelinckx I., Jans G., Voets W., Vanholsbeke C., Vansant G. (2014). Micronutrient levels and supplement intake in pregnancy afterbariatric surgery: A prospective cohort study. PLoS ONE.

[13] Osth M., Ost A., Kjolhede P., Stralfors P. (2014). The concentration of β-carotene in human adipocytes, but not the whole-body adipocyte stores, is reduced in obesity. PLoS ONE.

[14] Adams T.D., Hammoud A.O., Davidson L.E., Laferrère B., Fraser A., Stanford J.B., Hashibe M., Greenwood J.L.J., Kim J., Taylor D. (2015). Maternal and neonatal outcomes for pregnancies before and after gastric bypass surgery. Int. J. Obes..

[15] Shin D., Song W.O. (2015). Prepregnancy body mass index is an independent risk factor for gestational hypertension, gestational diabetes, preterm labor, and small-and large-for-gestational-age infants. J. Matern. Fetal. Neonatal..

[16] Narayanan R.P., Syed A.A. (2016). Pregnancy following bariatric surgery medical complications and management. Obes. Surg..

[17] Hamdy A.M., Abdel Aleem M.M., El-Shazly A.A. (2013). Maternal vitamin A deficiency during pregnancy and its relation with maternal and neonatal hemoglobin concentrations among poor Egyptian families. ISRN Pediatr..

[18] Mechanick J.L., Kusner R.F., Sugerman H.J., Gonzalez-Campoy J.M., Collazo-Clavell M.L., Spitz A.F., Apovian C.M., Livingston E.H., Brolin R., Sarwer D.B. (2009). American Association of Clinical Endocrinologists, The Obesity Society, and American Society for Metabolic and Bariatric Surgery medical guidelines for clinical practice for the perioperative nutritional, metabolic, and nonsurgical support of the bariatric surgery patient. Obesity.

[19] World Health Organization (1998). Obesity: Preventing and Managing the Global Epidemic.

[20] Institute of Medicine (IOM) (2009). Weight Gain during Pregnancy: Reexamining the Guidelines.

[21] Centers for Disease Control and Prevention (1998). Recommendations to prevent and control iron deficiency in the United States. MMWR Recomm. Rep..

[22] Sauberlich H.E., Hodges R.E., Wallace D.L., Kolder H., Canham J.E., Hood J. (1974). Vitamin A metabolism and requirements in the human studied with the use of labeled retinol. Vitam. Horm..

[23] World Health Organization (1996). Indicators for Assessing Vitamin A Deficiency and Their Application in Monitoring and Evaluating Intervention Programmes.

[24] Mclaren D.S., Frigg M. (1999). Manual de ver y Vivir Sobre los Transtornos por Deficiencia de Vitamina A (VADD).

[25] Saunders C., Leal M.C., Gomes M.M., Campos L.F.C., Dos Santos Silva B.A., Thiapó de Lima A.P.P., Ramalho R.A. (2004). Gestational nightblindness among women attending a public maternal hospital in Rio De Janeiro, Brazil. J. Health Popul. Nutr..

[26] Saunders C., Ramalho R.A., De Lima A.P.P.T., Gomes M.M., Campos L.F., Dos Santos Silva B.A., Gonçalves Soares A., Do Carmo Leal M. (2005). Association between gestational night blindness and serum retinol in mother/newborn pairs in the city of Rio de Janeiro, Brazil. Nutrition.

[27] Mecocci P., Polidori M.C., Troiand L., Cherubini A., Cecchetti R., Pini G., Marjanne S., Monti D., Stahl W., Sies H. (2000). Plasma antioxidants and longevity: A study on healthy centenarians. Free Radic. Biol. Med..

[28] World Health Organization (2014). Xerophthalmia and Night Blindness for the Assessment of Clinical Vitamin A Deficiency in Individuals and Populations.

[29] Machado S.N., Pereira S., Saboya C., Saunders C., Ramalho A. (2015). Influence of Roux-en-Y Gastric Bypass on the nutritional status of vitamin A in pregnant women: A comparative study. Obes. Surg..

[30] Christian P., West K.P., Khatry S.K., Katz J., Shrestha S.R., Pradhan E.K., Le Clerq S.C., Pokhrel R.P. (1998). Night blindness of pregnancy in rural Nepal—nutritional and health risks. Int. J. Epidemiol..

[31] Souza F.I.S., Amancio O.M.S., Sarni R.O.S., Pitta T.S., Fernandes A.P., Fonseca F.L.A., Hix S., Ramalho R.A. (2008). Non-alcoholic fatty liver disease in overweight children and its relationship with retinol serum levels. Int. J. Vitam. Nutr. Res..

[32] Villaça Chaves G., Pereira S.E., Saboya C.J., Ramalho A. (2008). Non-alcoholic fatty liver disease and its relationship with the nutritional status of vitamin A in individuals with class III obesity. Obes. Surg..

[33] Pereira S.E., Saboya C.J., Saunders C., Ramalho A. (2012). Serum levels and liver store of retinol and their association with night blindness in individuals with class III obesity. Obes. Surg..

[34] Tomedi L.E., Chang C.C., Newby P.K., Evans R.W., Luther J.F., Wisner K.L., Bodnar L.M. (2013). Pre-pregnancyobesityandmaternalnutritionalbiomarkerstatusduringpregnancy: A factor analysis. Public Health Nutr..

[35] Jeyakumar S.M., Vajreswari A. (2015). Vitamin A as a key regulator of obesity and its associated disorders: Evidences from an obese rat model. Indian. J. Med. Res..

[36] Jeyakumar S.M., Yasmeen R., Reichert B., Ziouzenkova O. (2013). Metabolism of vitamin A in white adipose tissue and obesity. Carotenoids and Vitamin A in Translational Medicine.

[37] Gillis L.J., Kennedy L.C., Bar-Or O. (2006). Overweight children reduce their activity levels earlier in life than healthy weight children. Clin. J. Sport. Med..

[38] Yasmeen R., Jeyakumar S.M., Reichert B., Yang F., Ziouzenkova O. (2011). The contribution of vitamin A to autocrine regulation of fat depots. Biochim. Biophys. Acta.

[39] Baugh N., Harris D.E., Aboueissa A.M., Sarton C., Lichter E. (2016). The impact of maternal obesity and excessive gestational weight gain on maternal and infant outcomes in Maine: Analysis of pregnancy risk assessment monitoring system results from 2000 to 2010. J. Pregnancy.

[40] Cañete A., Cano E., Muñoz-Chápuli R., Carmona R. (2017). Role of vitamin A/retinoic acid in regulation of embryonic and adult hematopoiesis. Nutrients.

[41] Benoist B., McLean E., Cogswell M., Egli I., Wojdyla D. (2008). Worldwide Prevalence of Anemia 1993–2005.

[42] Saltzman E., Karl J.P. (2013). Nutrient deficiencies after gastric bypass surgery. Annu. Rev. Nutr..

[43] Toh S.Y., Zarshenas N., Jorgensen J. (2009). Prevalence of nutrient deficiencies in bariatric patients. Nutrition.

[44] Moizé V., Deulofeu R., Torres F., Osaba J.M., Vidal J. (2011). Nutritional intake and prevalence of nutritional deficiencies prior to surgery in a Spanish morbidly obese population. Obes. Surg..

[45] Van Den Broek N. (2003). Anaemia and Micronutrient Deficiencies. Br. Med. Bull..

